# Radiomic Nomogram Improves Preoperative T Category Accuracy in Locally Advanced Laryngeal Carcinoma

**DOI:** 10.3389/fonc.2019.01064

**Published:** 2019-10-15

**Authors:** Fei Wang, Bin Zhang, Xiangjun Wu, Lizhi Liu, Jin Fang, Qiuying Chen, Minmin Li, Zhuozhi Chen, Yueyue Li, Di Dong, Jie Tian, Shuixing Zhang

**Affiliations:** ^1^Department of Radiology, The First Affiliated Hospital, Jinan University, Guangzhou, China; ^2^First Clinical Medical College, Jinan University, Guangzhou, China; ^3^CAS Key Laboratory of Molecular Imaging, Institute of Automation, Chinese Academy of Sciences, Beijing, China; ^4^College of Artificial Intelligence, University of Chinese Academy of Sciences, Beijing, China; ^5^State Key Laboratory of Oncology in South China, Collaborative Innovation Center for Cancer Medicine, Guangdong Key Laboratory of Nasopharyngeal Carcinoma Diagnosis and Therapy, Sun Yat-sen University Cancer Center, Guangzhou, China; ^6^Beijing Advanced Innovation Center for Big Data-Based Precision Medicine, School of Medicine, Beihang University, Beijing, China

**Keywords:** advanced laryngeal cancer, computed tomography, radiomics, T category, nomogram

## Abstract

Surgical decision-making on advanced laryngeal carcinoma is heavily depended on the identification of preoperative T category (T3 vs. T4), which is challenging for surgeons. A T category prediction radiomics (TCPR) model would be helpful for subsequent surgery. A total of 211 patients with locally advanced laryngeal cancer who had undergone total laryngectomy were randomly classified into the training cohort (*n* = 150) and the validation cohort (*n* = 61). We extracted 1,390 radiomic features from the contrast-enhanced computed tomography images. Interclass correlation coefficient and the least absolute shrinkage and selection operator (LASSO) analyses were performed to select features associated with pathology-confirmed T category. Eight radiomic features were found associated with preoperative T category. The radiomic signature was constructed by Support Vector Machine algorithm with the radiomic features. We developed a nomogram incorporating radiomic signature and T category reported by experienced radiologists. The performance of the model was evaluated by the area under the curve (AUC). The T category reported by radiologists achieved an AUC of 0.775 (95% CI: 0.667–0.883); while the radiomic signature yielded a significantly higher AUC of 0.862 (95% CI: 0.772–0.952). The predictive performance of the nomogram incorporating radiomic signature and T category reported by radiologists further improved, with an AUC of 0.892 (95% CI: 0.811–0.974). Consequently, for locally advanced laryngeal cancer, the TCPR model incorporating radiomic signature and T category reported by experienced radiologists have great potential to be applied for individual accurate preoperative T category. The TCPR model may benefit decision-making regarding total laryngectomy or larynx-preserving treatment.

## Background

Laryngeal cancer is a common malignant tumor in the head and neck and occurs mainly in smoking men (1). A study by the International Agency for Research on Cancer showed that 177,422 new laryngeal cancer cases occurred and resulted in 74,771 cancer-related deaths in 2018 (2).

The cancer control and functional outcomes of laryngeal cancer patients are highly relied on the treatment strategy. However, the management of laryngeal cancer remains controversial to date (3, 4). Currently, total laryngectomy is considered the most appropriate therapy for patients with advanced laryngeal carcinoma because they usually have a poor prognosis. Although total laryngectomy helps disease control, it has obvious adverse effects on patients' quality of life due to the loss of voice, permanent tracheostomy and issues with swallowing. In respect of which, Larynx-preserving surgery was thus performed to preserve laryngeal function (5). Decision-making about surgery are highly relied on tumor T category pursuant to the newest National Comprehensive Cancer Network (NCCN) Guidelines. The guidelines recommends total laryngectomy for all T4 stage and most of T3 stage laryngeal cancers, while some T3 stage diseases can benefit from larynx-preserving surgery instead (6).

Usually, the distinction between T3 and T4 categories is mainly based on the destruction degree of the extra-laryngeal spread and/or outer cortex of thyroid cartilage (7). However, accurate preoperative T category is clinically challenging. Currently, the most commonly used imaging techniques for T category (T3 vs. T4) are conventional imaging techniques including CT and MRI. CT generally demonstrates higher specificity but lower sensitivity as compared with MRI when identifying thyroid cartilage invasion (8). Although CT is useful in assessing the extent of extra-laryngeal spread or thyroid cartilage penetration of tumor, it has obvious limitations. Beitler et al. showed 74 and 81% positive predictive value of CT for assessing the extent of thyroid cartilage invasion and extra-laryngeal spread, respectively (9). However, Li et al. indicated that CT was less useful for assessing full-thickness cartilage invasion, with 47% of T4 disease being down-staged to T3 disease after pathological review (10). In contrast, MRI is more sensitive than CT in detecting cartilage invasion, yet peritumoral inflammation, edema and fibrosis may demonstrate similar features with cartilage invasion (11). These findings indicated the difficulty of accurate T category before surgery. Therefore, to develop new non-invasive methods for preoperative evaluation are needed for the purpose of determining the extent of extra-laryngeal spread and thyroid cartilage penetration, which are the most important considerations for selecting total laryngectomy or larynx conservation.

In recent years, the proposed “radiomics” is developed rapidly and has attracted great attention. It aims to extract huge amounts of objective features from medical images and find out the significant features which have great potential to expose disease characteristics that failed to be discovered by naked eyes (12–15). Previous studies showed that radiomic signatures as biomarkers have close correlations with clinical stages, lymph node metastasis, and survival outcomes (16–19). As there is no study explored whether radiomics would enhance the accuracy of preoperative T category for patients with advanced laryngeal cancer, we tried to explore CT-based TCPR as a novel approach for individual accurate preoperative T category for those patients, which would benefit clinical decision-making (total laryngectomy or larynx conservation) before surgery.

## Materials and Methods

### Patient Population

This retrospective study was approved by the Institutional Review Board and the informed consent requirement was waived. The whole cohort of this study was acquired from the medical records of the Institutional database from April 2007 to March 2015. Patients with histologically confirmed laryngeal cancer who had received total laryngectomy were included. Contrast-enhanced CT examinations of the neck had been performed on all patients before surgery. The inclusion criteria were as follows: (1) newly diagnosed patients underwent contrast-enhanced CT scans of neck before any treatment; (2) patients received total laryngectomy 15 days after initial CT acquisition; and (3) patients had pathologically confirmed T3 or T4 stage laryngeal cancer after operation. The exclusion criteria were as follows: (1) poor quality of CT images due to patients' movement or artifacts, etc.; (2) the slice thickness of CT scan >2.5 mm; and (3) patients received treatment.

A total of 211 patients met these criteria. Among which, 150 patients constituted the training cohort, including 146 males and four females with mean age of 61.38 ± 8.54 ranging from 39 to 85. A total of 61 patients (59 males, two females) with mean age of 60.23 ± 6.65 ranging from 30 to 78 were allocated to the validation cohort.

**Figure 1:**
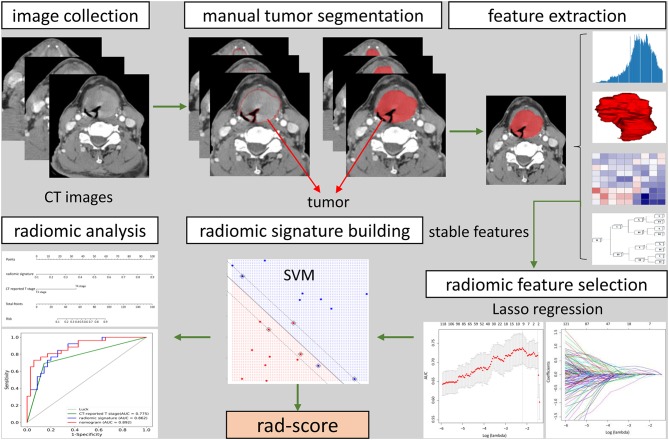
The workflow of radiomic analysis in the current study. After feature extraction, stable features were selected by LASSO for further analysis. SVM model was used to build radiomic signature. The predictive nomogram was constructed based on the radiomic signature and other predictors.

Clinicopathologic data was collected from the medical records and the data of baseline CT scans, including age, gender, preoperative T category reported by head and neck radiologists, and pathologically confirmed T category. T classification was conducted pursuant to the 8th Edition of AJCC TNM Staging System Guidelines (20), and then reassessed by a head and neck radiologist with 20 years of experience who was blinded to the pathology results. Figure 1 showed the workflow of radiomic analysis in the current study.

### CT Image Acquisition and Tumor Segmentation

Two CT systems were adopted for CT image acquisition: United Imaging uCT780 and Siemens SOMATOM Force CT. The parameters for CT image acquisition were as follows: 110–120 kV; 116–168 mAs; detector collimation: 192 × 0.6 mm or 160 × 0.25 mm; rotation time: 1.0 s; slice thickness: 1–2.5 mm; field of view: 250 × 250 mm; matrix: 512 × 512.

Axial venous phase CT images (DICOM format) were prepared for tumor segmentation. An open-source software ITK-SNAP (www.itk-snap.org) was applied for the three dimensional manual segmentation. Tumor region in each layer was outlined by a radiologist with 12 years of experience in head and neck cancer, and then validated by a senior radiologist with 20 years of experience in head and neck cancer. The regions of interest covering the entire tumor were used for subsequent feature extraction.

### Radiomic Features Extraction and Radiomic Signature Construction

Radiomic features were extracted by Pyradiomics (version 2.1.2), an open-source python platform (http://www.radiomics.io/pyradiomics.html) (21). Pyradiomics provides a stably operated open-source platform for easy and reproducible radiomic features extraction that can be compared across different institutions. Features of high throughput were extracted from CT images by matrix operation, wavelet transform and other mathematical methods, whose purpose was to find out the association between radiomic features and pathologically confirmed T category. The extracted radiomic features were classified into 4 categories: first-order features (*n* = 126), textural features (*n* = 515), shape-based features (*n* = 13), wavelet features (*n* = 736). In order to identify the most significant features, we used the interclass correlation coefficient and least absolute shrinkage and selection operator (ICC-LASSO) to remove abundant high dimensional features. Only features with an ICC > 0.75 were retained for further LASSO, while the remaining radiomic features were excluded to ensure the stability and reproducibility. After that, the most significant features were used to build the support vector machine (SVM) machine learning prediction model. Grid search and cross validation were conducted to select model parameters, which optimize the performance of the model. Then, radiomic signature was obtained from the trained SVM model.

### Diagnostic Validation of Radiomic Signature

We used AUC, sensitivity, specificity, and accuracy to verify the association between radiomic signature and pathologically confirmed T category in order to determine the overall performance of the model. The performance of radiomic signature was established in the training cohort and internally validated in the validation cohort.

### Development of an Individualized T Category Prediction Nomogram

Univariate analysis was performed on clinical features, such as age, gender, tumor location, and T category reported by an experienced radiologist. The most significant clinical features and radiomic signature were combined to establish a multivariable logistics model so as to develop a radiomic nomogram.

### Validation of the Radiomic Nomogram

The utility of the radiomic nomogram in the training and validation cohorts was assessed by the receiver operating characteristic (ROC) curves. The ROC curve was plotted basing on the predictors of multi-logistics model including AUC, sensitivity, specificity, and accuracy. In addition, we plotted the calibration curves and conducted the Hosmer-Lemeshow test to demonstrate the calibration of the radiomic nomogram.

### Statistical Analysis

Continuous data were presented as mean ± standard deviation (SD), while categorical data were presented as counts and percentages. Continuous and categorical data were compared by independent t (or Mann-Whitney U) test and Chi-square (or Fisher's exact) statistics, respectively. Patients were randomly divided into the training and validation cohorts at a ratio of ~2.5:1. The average performance of the model was obtained by bootstrapping for 2,000 times. All statistical analyses were conducted by R software (version 3.5.1) and Python (version 3.6). The R software was used for features selection and building nomogram with packages of “psych,” “glmnet,” and “rms,” while the Python was used to build SVM model with “sklearn” package.

## Results

### Clinical Characteristics

Table 1 summarizes the patient characteristics of the training and validation cohorts. Only T category reported by radiologists showed significant difference (*P* < 0.001). After pathological review, 20.5% (17/83) of patients down-staged from T4 to T3, and 28.9% (37/128) of patients over-staged from T3 to T4.

**Table 1:** Patient characteristics in the training and validation cohorts.

**Characteristics**	**Training cohort (*****n*** **= 150)**			**Validation cohort (*****n*** **= 61)**		
	**T3 category**	**T4 category**	***P***	**T3 category**	**T4 category**	***P***
Gender, No (%)			0.343			0.501
Male	70 (95.9%)	76 (98.7%)		33 (94.3%)	26 (100%)	
Female	3 (4.1%)	1 (1.3%)		2 (5.7%)	0	
Age, mean ± SD, years	61.38 ± 8.54	63.72 ± 8.97	0.157	60.23 ± 6.65	60.31 ± 10.91	0.737
Location, No (%)			0.022			0.579
Supra-glottis	31 (42.5%)	21 (27.3%)		11 (31.4%)	10 (38.5%)	
Glottis	40 (54.8%)	56 (72.7%)		24 (68.6%)	16 (61.5%)	
Sub-glottis	2(2.7%)	0		0	0	
T category reported by radiologist, No (%)			<0.001			<0.001
T3 category	61 (83.6%)	29 (37.7%)		30 (85.7%)	8 (30.8%)	
T4 category	12 (16.4%)	48 (62.3%)		5 (14.3%)	18 (69.2%)	

### Radiomic Features Extraction and Radiomic Signature Construction

**Figure 2:**
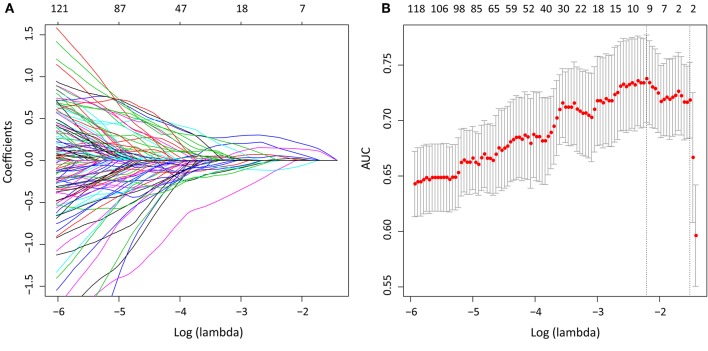
After initial screening by ICC analysis, feature selection was performed using the LASSO method with a logistic regression model. **(A)** The model coefficient trendlines of the 1,390 radiomics features. The profile graph was plotted by coefficients against the L1 norm (inverse proportional to log λ = −2.184). **(B)** Tuning parameter λ in the LASSO model. The parameter λ = 0.220 were selected under the minimum criteria. The vertical line was drawn at the value selected by 10-fold cross-validation, including optimized eight non-zero coefficients.

We extracted 1,390 features in total from CT images, among which, 565 had ICC > 0.75, which indicted a good inter-measurer agreement. LASSO was then used to remove the redundancy of high dimensional features, and eight significant radiomic features were selected at last (Figure 2), including two first order features (gradient_first order_Skewness, lbp.2D_first order_Mean), two shape features (original_shape_LeastAxis, original_shape_Sphericity), and four wavelet features (wavelet-LLH_first order_Kurtosis, wavelet-LLH_glcm_Idn, wavelet-LLH_first order_Median, wavelet-LLL_glcm_Imc1). A SVM-based radiomic signature was constructed based on the eight features.

### Diagnostic Validation of Radiomic Signature

The AUCs of radiomic signature were 0.850 (95% CI: 0.788–0.912) and 0.862 (95% CI: 0.772–0.952) in the training and validation cohorts, respectively (Table 2). Correspondingly, the specificity were 0.792 (95% CI: 0.698–0.885) and 0.743 (0.598–0.888); the sensitivity were 0.782 (95% CI: 0.690–0.874) and 0.808 (95% CI: 0.656–0.959); and the accuracy were 0.787 (95% CI: 0.784–0.789) and 0.770 (95% CI: 0.765–0.776) (Table 2).

**Table 2:** Diagnostic performance of models in the training and validation cohorts.

**Models**	**Training cohort (*****n*** **= 150)**				**Validation cohort (*****n*** **= 61)**			
	**AUC (95% CI)**	**Specificity (95% CI)**	**Sensitivity (95% CI)**	**Accuracy (95% CI)**	**AUC (95% CI)**	**Specificity (95% CI)**	**Sensitivity (95% CI)**	**Accuracy (95% CI)**
T category reported by radiologist	0.751 (0.684–0.818)	0.861 (0.781–0.941)	0.641 (0.535–0.747)	0.747 (0.744–0.749)	0.775 (0.667–0.883)	0.857 (0.741–0.973)	0.692 (0.515–0.870)	0.787 (0.781–0.792)
Radiomic signature	0.850 (0.788–0.912)	0.792 (0.698–0.885)	0.782 (0.690–0.874)	0.787 (0.784–0.789)	0.862 (0.772–0.952)	0.743 (0.598–0.888)	0.808 (0.656–0.959)	0.770 (0.765–0.776)
Combined nomogram	0.899 (0.850–0.947)	0.889 (0.816–0.961)	0.782 (0.690–0.874)	0.833 (0.832–0.835)	0.892 (0.811–0.974)	0.771 (0.632–0.911)	0.808 (0.656–0.959)	0.787 (0.781–0.792)

### Development of an Individualized T Category Prediction Nomogram

**Figure 3:**
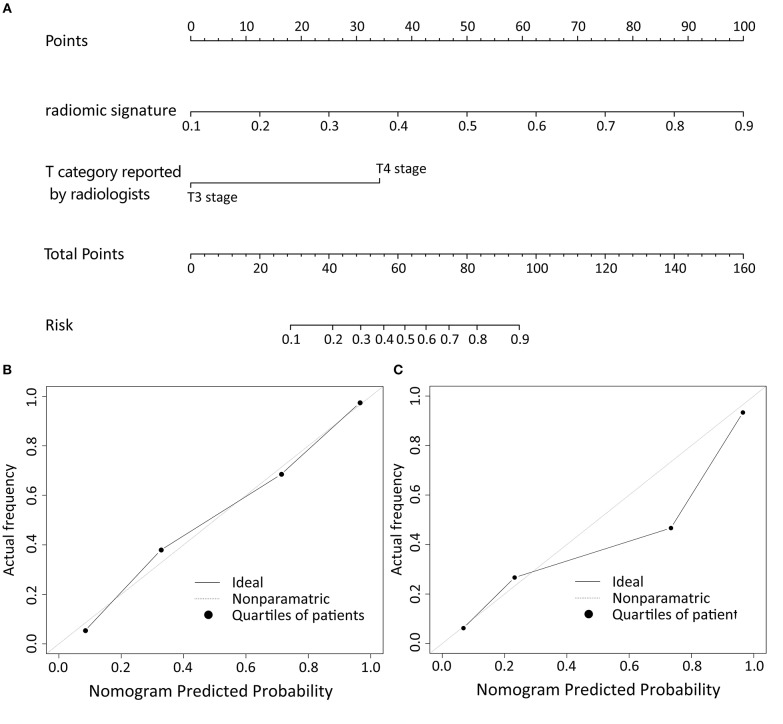
The nomogram of T category diagnostic model. Our radiomics based nomogram was constructed in the training cohort. The radiomic signature, T category reported by radiologist were incorporated as factors **(A)**. The calibration curves showed good agreement between the nomogram-predicted T category and actual T category in the training cohort **(B)** and validation cohort **(C)**.

Logistic regression analysis of clinical features demonstrated that only T category reported by experienced radiologist was significantly correlated with pathologically confirmed T category (*P* < 0.001). Radiomic nomogram was established by combining radiomic signature and T category reported by radiologists (Figure 3A). The calibration curves of nomogram showed a good agreement between prediction and observation in both of the training and validation cohorts (Figures 3B,C).

### Validation of the Radiomic Nomogram

In the training cohort, the AUC of T category reported by radiologists was 0.751 (95% CI: 0.684–0.818), with specificity of 0.861 (95% CI: 0.781–0.941), sensitivity of 0.641 (95% CI: 0.535–0.747), and accuracy of 0.747 (95% CI: 0.744–0.749) (Table 2). The AUC of the combined nomogram incorporating radiomic signature and T category reported by radiologists was 0.899 (95% CI: 0.850–0.947), with sensitivity of 0.782 (95% CI: 0.690–0.874), specificity of 0.889 (95% CI: 0.816–0.961), and accuracy of 0.833 (95% CI: 0.832–0.835) (Table 2).

**Figure 4:**
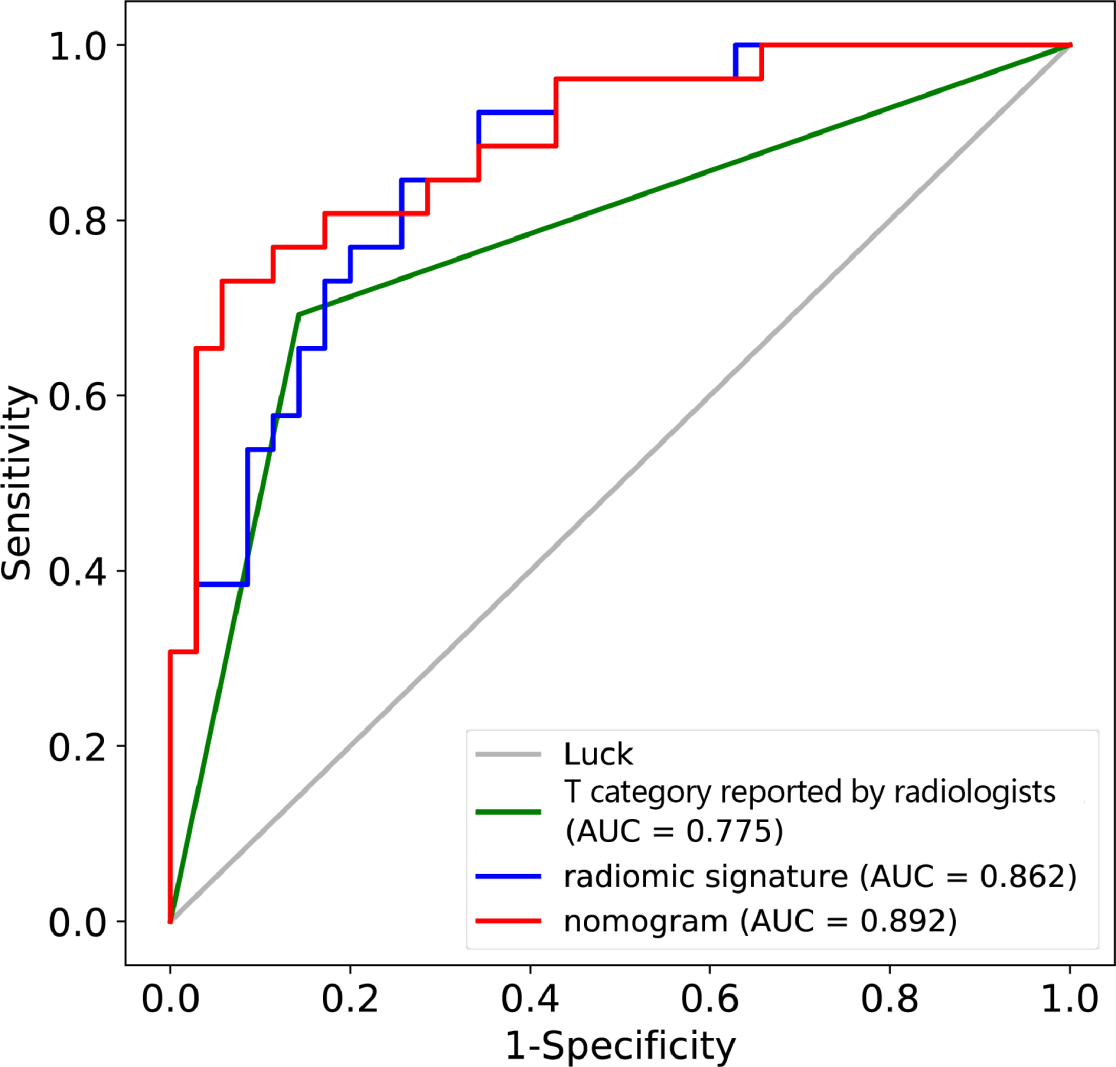
ROC curves for the nomogram, radiomic signature, and T category reported by radiologist in the training and validation datasets.

In the validation cohort, the AUC of T category reported by radiologists was 0.775 (95% CI: 0.667–0.883) with specificity of 0.857 (95% CI: 0.741–0.973), sensitivity of 0.692 (95% CI: 0.515–0.870), and accuracy of 0.787 (95% CI: 0.781–0.792) (Table 2, Figure 4). The AUC of the nomogram incorporating radiomic signature and T category reported by radiologists was 0.892 (95% CI: 0.811–0.974), with sensitivity of 0.808 (95% CI: 0.656–0.959), specificity of 0.771 (95% CI: 0.632–0.911), and accuracy of 0.787 (95% CI: 0.781–0.792) (Table 2, Figure 4).

## Discussion

This study developed and validated a radiomic nomogram for the accurate prediction of T category (T3 vs. T4) before surgery for patients with locally advanced laryngeal cancer. The combined nomogram incorporated the CT-reported T stage and the radiomic signature. By only CT, radiologists couldn't satisfactorily stratified patients into T3 and T4 categories (AUC = 0.775). However, the combination of the radiomic signature and the T category reported by radiologists could significantly improve the predictive performance, achieving an AUC of 0.892 in the validation cohort.

Locally advanced laryngeal cancer includes those classified as T3 or T4 category (22). For locally advanced laryngeal cancer, the treatment option of total laryngectomy or organ preservation remains to be a hot-debated topic. The goal of larynx preservation is to achieve good function without altering patients' survival. When determining larynx preservation or total laryngectomy for patients, some issues must be considered, such as T category of tumor, patients' will, and prospects for a good functional outcome (23). Therefore, preoperative T category is particularly important. If we could distinguish T3 from T4 patients with laryngeal cancer, they can receive appropriate treatment and benefit a lot. This study is focused on patients with local advanced laryngeal cancer and tried to find out a new method to distinguish T3 from T4 disease accurately.

Previous studies demonstrated that CT, MRI, PET-CT images can reflect the tumor morphology (24–26). Clinicians rely on medical imaging to determine whether patients suffered from T3 or T4 disease. Reliable imaging tools are indispensable. CT is the preferred imaging method for laryngeal cancer staging (11), which is much faster than MRI. MRI has better discrimination of soft tissue changes and cartilage abnormalities, however, it requires longer image acquisition time, thus challenging patients' cooperation and hampering its utilization (27). Still, it is very important for imaging techniques being able to differentiate inner cortical invasion (T3) from destruction of the outer cortex and extra-laryngeal spread. The evaluation on thyroid cartilage invasion and extra-laryngeal spread is important and sometimes difficult, and the positive predictive value of CT-reported T category is 71.1%, similar with Li et al. (10). MRI seems to be more sensitive than CT in detecting cartilage invasion, however, the MRI findings are not specific, and the positive predictive value of MRI was unsatisfactory (9). This is because that peritumoral inflammation, edema and fibrosis may demonstrate similar features with cartilage invasion (11). Currently, the guidelines recommends total laryngectomy for patients with T4 stage diseases, while for T3 stage diseases, organ preservation or total laryngectomy are all listed options (6). It is indicated that some patients who were treated by total laryngectomy could have been offered laryngeal preservation or who received laryngeal preservation actually need total laryngectomy to extend the survival time if more accurately staging was performed at initial diagnosis (10, 28).

Radiomics is a new concept in recent years, and it is gaining importance in cancer research for improving diagnostic, prognostic, and predictive accuracy (29). Zhang et al. established and internally validated MRI-derived radiomics as a new approach to evaluate progression-free survival in patients with stage III–IVb nasopharyngeal carcinoma before treatment (30). Liang et al. demonstrated that a combined nomogram model could preoperatively predict histologic grade in patients with pancreatic neuroendocrine tumors (18). For patients with locally advanced laryngeal cancer, we identified a radiomic nomogram to perform preoperative predicting of tumor T category (T3 vs. T4). To construct a radiomic signature, 1,382 (99.4%) radiomic features were filtered, and only eight features were saved by ICC and LASSO analysis. For huge amounts of radiomic features extracted from a relatively small sample, LASSO can avoid model overfitting (31). In addition, the features selected by LASSO are generally accurate and can be easily interpreted because the vast majority of irrelevant features' coefficients are shrunk toward zero during model fitting. The radiomic signature was constructed through LASSO-SVM by combining radiomic features with other clinical features, such as age, gender, tumor location, T category reported by radiologists. Our study showed that preoperative T category reported by radiologists was easily obtained and significantly correlated with actual T category, and it might significantly influence the accuracy of the prediction of T category. Therefore, the radiomic nomogram incorporates both the T category reported by radiologists and the radiomic signature to ensure accuracy. The T category reported by radiologists can stratify patients into T3 and T4 groups with an AUC of 0.751. However, the combined nomogram model can further improve the predictive performance, achieving an AUC of 0.899. This prediction model was also tested by the validation cohort (AUC = 0.892), verifying the reliability and reproducibility.

The main limitation of this study derived from its retrospective nature. To keep the consistency of data, the training and validation cohorts were from a single institution. When determining the most suitable treatment strategy for advanced laryngeal cancer, preoperative T category is not the only factor under consideration, other conditions should also be considered, such as tumor volume, lymphatic metastasis, pre-treatment voice quality, laryngoscopy findings, patient comorbidities, and preferences.

In conclusion, this study established a TCPR model as a novel approach for the accurate preoperative T category for patients with locally advanced laryngeal cancer. As a non-invasive, preoperative and precise T category evaluation tool, the model could assist head and neck surgeons to make an appropriate surgical decision, which will benefit patients in the future.

## Data Availability Statement

The raw data supporting the conclusions of this manuscript will be made available by the authors, without undue reservation, to any qualified researcher.

## Ethics Statement

The studies involving human participants were reviewed and approved by The First Affiliated Hospital, Jinan University. Written informed consent for participation was not required for this study in accordance with the national legislation and the institutional requirements.

## Author Contributions

FW, BZ, and SZ: conception and design. FW, LL, JF, ML, ZC, YL, and QC: acquisition of data. XW, DD, and JT: analysis and interpretation of data. FW, BZ, DD, JT, and SZ: drafting or revising the article.

### Conflict of Interest

The authors declare that the research was conducted in the absence of any commercial or financial relationships that could be construed as a potential conflict of interest.

## Abbreviations

TCPR: T category prediction radiomics
LASSO: least absolute shrinkage and selection operator
AUC: area under the curve
ICC: interclass correlation coefficient
ROC: receiver operating characteristic
SVM: support vector machine.
